# Anomalous Features of EMT during Keratinocyte Transformation

**DOI:** 10.1371/journal.pone.0001574

**Published:** 2008-02-06

**Authors:** Tamar Geiger, Helena Sabanay, Nataly Kravchenko-Balasha, Benjamin Geiger, Alexander Levitzki

**Affiliations:** 1 Department of Biological Chemistry, Institute of Life Science, The Hebrew University, Jerusalem, Israel; 2 Department of Molecular Cell Biology, Weizmann Institute of Science, Rehovot, Israel; University of Oldenburg, Germany

## Abstract

During the evolution of epithelial cancers, cells often lose their characteristic features and acquire a mesenchymal phenotype, in a process known as epithelial-mesenchymal transition (EMT). In the present study we followed early stages of keratinocyte transformation by HPV16, and observed diverse cellular changes, associated with EMT. We compared primary keratinocytes with early and late passages of HF1 cells, a cell line of HPV16-transformed keratinocytes. We have previously shown that during the progression from the normal cells to early HF1 cells, immortalization is acquired, while in the progression to late HF1, cells become anchorage independent. We show here that during the transition from the normal state to late HF1 cells, there is a progressive reduction in cytokeratin expression, desmosome formation, adherens junctions and focal adhesions, ultimately leading to poorly adhesive phenotype, which is associated with anchorage-independence. Surprisingly, unlike “conventional EMT”, these changes are associated with reduced Rac1-dependent cell migration. We monitored reduced Rac1-dependent migration also in the cervical cancer cell line SiHa. Therefore we can conclude that up to the stage of tumor formation migratory activity is eliminated.

## Introduction

Epithelial-mesenchymal transition (EMT) is a developmental process characterized by the loss of cell-cell adhesion, leading to cell individualization, which is accompanied by increased cell motility. Cancer development, and the initiation of invasion and metastasis, which involves dissemination of cells to other tissues has many EMT features. The most prominent phenotypic change associated with keratinocyte transformation is the reduction in cell-cell adhesion. Normal keratinocytes form a multi-layered sheet, held together by robust cell-cell adhesions, dominated by dense arrays of actin-associated adherens junctions and cytokeratin-bound desmosomes. During EMT, these intercellular junctions are down-regulated, leading to the loss of epithelial coherence [1]–[4]. Adherens junctions connect epithelial cells through homotypic interactions mediated by E-Cadherin molecules. E-cadherin cytoplasmic domains, connect to the actin cytoskeleton through the catenin protein family, namely beta-catenin, alpha-catenin and p120-catenin. Down-regulation of E-cadherin is a hallmark of EMT, which often coincides with the accumulation of beta-catenin in the nucleus and activation of its target genes [4]. Desmosomes consist of desmosomal cadherins, namely, desmogleins and desmocollins, which connect to the intermediate filament network through a complex of adaptor proteins such as plakoglobin, plakophilin and desmoplakin [5]. A reduction in desmosome formation during EMT correlates with conspicuous alterations in intermediate filaments; cytokeratins, almost disappear from within the cell, while the mesenchymal-type intermediate filament protein, vimentin, is up-regulated [2], [6]–[8]. Apart from separation of cell-cell adhesion, cell-individualization also results from reduction in extra-cellular matrix (ECM) adhesion. Furthermore, EMT is commonly associated with increased cell migration, which enables cells to dissociate from their original tissue and form metastasis in distant organs. Increased migratory capacity often depends on Rac1 activity, which induces lamellipodia formation and focal complex assembly [9].

Human Papillomaviruses (HPV) are implicated in cervical cancer and other ano-genital tumors. High-risk HPVs, mainly types 16, 18, 31 and 33 are the cause of >90% of cervical cancers. Infection of keratinocytes with these HPV types in vivo, immortalizes the cells and induces a dysplastic phenotype through distortion of the stratified structure of the epithelium and reduction in tissue differentiation [10], [11], which are associated with the down-regulation of E-cadherin, desmosomes and cytokeratins [12]–[14], characteristic of EMT.

We have recently described an in-vitro model for cervical cancer development, using the continuous passaging of HPV 16-immortalized human keratinocytes [15], [16]. Here we have combined molecular biology, immuno-fluorescence microscopy, time-lapse video microscopy and electron microscopy to study the cellular features of pre-cancerous stages of HPV16-induced transformation. We show that preeminent features of EMT already take place prior to full transformation of the HPV16 immortalized keratinocytes. We compared a cell line of HPV16 transformed keratinocytes, HF1 cells, from various stages, to their normal counterparts, namely, uninfected primary keratinocytes. Early HF1 cells are ∼60 doublings post transfection and late HF1 cells are ∼1000 doublings post transfection of HPV16. Late HF1 cells have a high proliferation rate, form small colonies in soft agar [16], and show many overlapping features in gene expression with cervical cancers (Kravchenko-Balasha et al, submitted), however they do not form tumors in nude mice, and are therefore considered to be in an advanced stage of the pre-cancerous phase. In order to examine the generality of our observations we studied also the cervical cancer cell line SiHa. We show that key features of EMT occur during the continuous transition from primary keratinocytes to late HF1 cells. These features include reductions in cytokeratin expression, desmosome formation, adherens junction and focal adhesion assembly. However, concomitantly, late HF1 cells as well as SiHa cells show dramatically reduced Rac1-dependent migration.

## Results

In this study we used HPV16-transformed keratinocytes in an attempt to explore the cellular changes that occur at early stages of transformation. We compared primary keratinocytes to various stages of HPV16 transformed cell-line, HF1 cells. Early and late HF1 cells are ∼60 and ∼1000 cell doublings after transfection of the HPV16 genome, respectively. We explored parameters of EMT including cytoskeletal proteins, cell adhesion and migration, and examined their changes in early stages of transformation.

### Cytokeratin status

**Figure 1:**
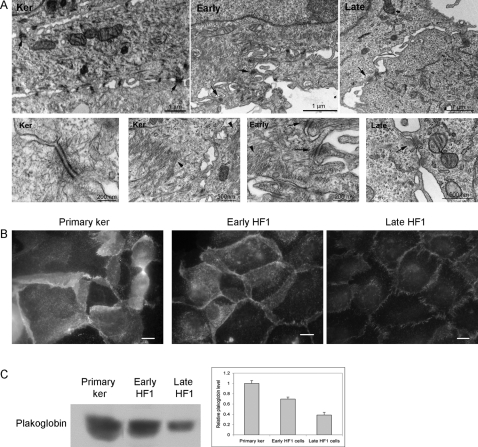
Desmosomes and cytokeratin are gradually reduced in the course of transformation. A. Primary keratinocytes, early and late HF1 cells were examined by transmission electron microscopy (TEM). Arrows show representative desmosomes, and arrowheads point to areas of cytokeratin bundles. B. Representative immuno-fluorescence images of plakoglobin staining. Bar = 15 µm. C. Western blot analysis of plakoglobin expression in the primary keratinocytes, early and late HF1 cells. Graph shows quantification of three experiments.

The pattern of cytokeratin expression is a marker for the degree of cell differentiation and transformation [6]. It was shown that during EMT, cytokeratin levels are reduced, while vimentin, the mesenchymal intermediate filament species is elevated [2], [6], [7], [17], [18]. We monitored decreased abundance of intermediate filaments at early stages of transformation by transmission electron microscopy (TEM). In the primary keratinocytes we observed massive distribution of cytokeratin bundles throughout the cell, while early HF1 cells had a delicate network of intermediate filaments, which was further reduced upon progression to late HF1 cells (Figure 1A).

Using the Affymetrix Human Genome microarrays, we compared gene expression in each of these cell types. We monitored a marked reduction in cytokeratin 1 and 16, which are typical of stratified epithelium, in early HF1 cells compared to the primary keratinocytes, with smaller, but significant reductions in cytokeratins 4, 15 and 75 (Table 1). More dramatic reductions were observed after progression to late HF1 cells, which expressed less than 20% of mRNA encoding cytokeratins 1, 4, 13, 15, 16, 23, 24 and 75, compared to the primary keratinocytes. Expression of cytokeratins 18 and 19, typical of simple, non-stratified normal or malignant epithelia [6], was increased 4-fold, in both early and late HF1 cells. The level of vimentin mRNA was 1.8 higher in late HF1 cells than in the primary keratinocytes (Table 1).

**Table 1:** Intermediate filament expression

	Ker	Early HF1	Late HF1
Cytokeratin 1	1±0.2	0.0±0.0	0.0±0.0
Cytokeratin 2	1±0.6	1.4±1.2	0.8±0.3
Cytokeratin 3	1±0.5	0.6±0.2	0.4±0.0
Cytokeratin 4	1±0.3	0.5±0.1	0.0±0.0
Cytokeratin 5	1±0.1	1.0±0.1	1.1±0.1
Cytokeratin 6A	1±0.1	0.9±0.2	0.7±0.3
Cytokeratin 6B	1±0.1	0.8±0.2	0.6±0.3
Cytokeratin 6C	1±0.1	1.0±0.1	0.9±0.0
Cytokeratin 7	1±0.1	0.7±0.2	0.4±0.2
Cytokeratin 9	1±0.2	0.9±0.3	1.2±0.2
Cytokeratin 10	1±0.2	0.8±0.1	1.6±0.2
Cytokeratin13	1±0.2	1.4±0.2	0.1±0.0
Cytokeratin14	1±0.1	1.0±0.2	1.0±0.0
Cytokeratin 15	1±0.1	0.4±0.1	0.0±0.0
Cytokeratin 16	1±0.2	0.2±0.0	0.0±0.0
Cytokeratin 17	1±0.1	0.9±0.1	0.7±0.2
Cytokeratin18	1±0.5	3.6±0.7	3.9±0.6
Cytokeratin19	1±0.7	5.4±0.5	3.9±0.6
Cytokeratin 23	1±0.5	0.6±0.5	0.1±0.0
Cytokeratin 24	1±0.6	4.1±0.2	0.2±0.1
Cytokeratin 75	1±0.2	0.3±0.0	0.2±0.1
Vimentin	1±0.1	0.1±0.0	1.8±0.1
GAPDH	1±0.1	1.0±0.1	1.1±0.1

Expression of intermediate filaments: cytokeratin and vimentin according to Affymetrix Human Genome U133A microarrays. Results were normalized to the expression in the primary keratinocytes. GAPDH expression is given as internal control. Standard deviations are of three arrays for each cell type.

### Loss of desmosomes, adherens junctions and focal adhesions

Examination of cells by TEM clearly showed that the primary keratinocytes were highly adherent, forming a confluent sheet of cells, extensively connected through dense plaque containing desmosomes. Desmosomes were slightly reduced in early HF1 cells, and more dramatically in late HF1 cells, where we could not detect desmosomes and only occasional adherens junctions were seen (Figure 1A). Concomitantly, the apparent distance between the cells was markedly increased in late HF1 cells, compared to the primary keratinocytes and early HF1 cells. To further characterize the reduction in cell-cell adhesion following HPV16 transformation, we labeled cells for plakoglobin, an adaptor protein, which links desmosomal cadherins to the cytokeratin network, and, more rarely, resides in adherens junctions. Immunostaining of plakoglobin was very intense in the primary keratinocytes, and progressively reduced in early and in late HF1 cells, where it was barely detectable (Figure 1B). Furthermore, while in the primary keratinocytes junctions were very wide and encompassed large areas of interdigitated cellular protrusions, junctional areas were markedly reduced already in early HF1 cells. Western blot analysis confirmed the progressive decrease in plakoglobin protein expression from the primary keratinocytes through early HF1 cells to late HF1 cells (Figure 1C).

**Figure 2:**
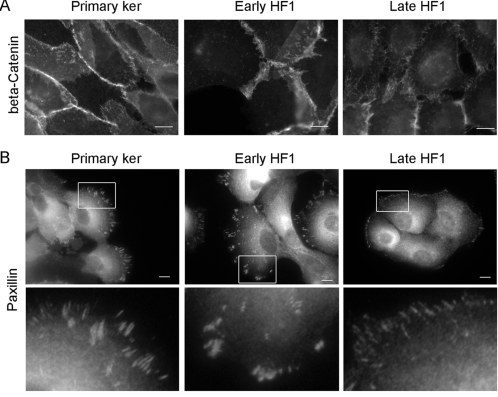
Representative immuno-fluorescence images of primary keratinotyes, early and late HF1 cells. A. Staining with anti-beta-Catenin antibody. B. Staining with anti-Paxillin antibody. Bar = 15 µm. Enlargements of focal adhesions are shown below.

Down-regulation of desmosomes during transformation is at least partially attributable to reduced mRNA expression of desmosomal genes. Affymetrix cDNA results revealed a ∼10-fold reduction in the expression of the desmosomal cadherins: desmocollins and desmogleins, and in periplakin in late HF1 cells as compared to the primary keratinocytes (Table 2). Significant, changes were also observed in other desmosomal components, including plakoglobin, plakophillin and envoplakin. These changes were already observed in early HF1 cells, and correlated well with the apparent changes in the formation of desmosomes and cytokeratin filaments. The changes observed in adherens junctions were more modest. Our microarray data showed a 2-fold decrease in E-cadherin expression in late HF1 cells, compared to the primary keratinocytes (Table 2), and no change in the levels of other adherens junction components, including alpha and beta-catenin and P-cadherin. Immuno-staining of cells with anti-beta-catenin antibody showed intense staining of the primary keratinocytes, over large overlapping areas at the cell periphery, similar to plakoglobin staining (Figure 2A). This apparent overlap was reduced in early HF1 cells, and completely abolished in late HF1 cells. Nevertheless, most of the beta-catenin was still concentrated in cell-cell junctions, and no significant translocation of beta-catenin to the nucleus could be detected. Reduced cell-cell adhesion coincided with the partial dissociation of cells from the ECM. Upon monitoring of focal adhesions/complexes by paxillin staining, we noted a small decrease in focal adhesion number in early HF1 cells, compared to the primary keratinocytes, and a more dramatic decrease in the progression to late HF1 cells (Figure 2B).

**Table 2:** Expression of desmosomal and adherens junction genes

	Ker	Early HF1	Late HF1
Desmocollin1	1±0.6	0.1±0.1	0.1±0.1
Desmocollin2	1±0.5	0.3±0.1	0.0±0.0
Desmocollin3	1±0.1	0.6±0.1	0.2±0.1
Desmoglein1	1±0.5	0.0±0.0	0.0±0.0
Desmoglein2	1±0.0	1.6±0.1	0.6±0.1
Desmoglein3	1±0.1	0.5±0.2	0.1±0.0
Plakoglobin	1±0.1	0.8±0.1	0.5±0.1
Plakophillin1	1±0.2	0.4±0.2	0.6±0.4
Plakophillin2	1±0.2	0.8±0.2	0.4±0.1
Plakophillin3	1±0.1	1.0±0.2	0.7±0.1
Desmoplakin	1±0.1	0.7±0.1	0.7±0.1
Plectin	1±0.2	1.7±0.6	1.0±0.0
Envoplakin	1±0.3	0.8±0.1	0.3±0.1
Periplakin	1±0.2	0.7±0.2	0.1±0.0
alpha-Catenin	1±0.1	0.8±0.0	0.7±0.1
Beta-Catenin	1±0.0	1.0±0.1	1.1±0.1
E-Cadherin	1±0.2	0.9±0.1	0.5±0.2
P-Cadherin	1±0.1	1.6±0.1	0.9±0.1
GAPDH	1±0.1	1.0±0.1	1.1±0.1

Expression of desmosomal and adherens junction genes according to Affymetrix Human Genome U133A microarrays. Results were normalized to the expression in the primary keratinocytes. GAPDH expression is given as internal control. Standard deviations are of three arrays for each cell type.

### Transcription factors

Interestingly, all parameters examined so far showed that hallmarks of EMT were apparent in cells shortly after HPV16 introduction, and that EMT progressed significantly in the precancerous stage. This notion was also supported by quantification of the expression of several transcription regulators that are considered essential for the initiation and progression of EMT. While ZEB1 showed a 2-fold decrease in expression, Slug and ZEB2 expression were almost 2-fold higher in late HF1 cells compared to the primary keratinocytes, and showed even higher induction compared to early HF1 cells (Table 3). Interestingly, TWIST expression, which was mostly implicated in the formation of metastases [19]–[21], was significantly elevated (∼4.5-fold) following the transition from the primary keratinocytes to early HF1 and even more dramatically in late HF1 cells (∼13-fold).

**Table 3:** Expression of transcriptional regulators of EMT

	Primary ker	Early HF1	Late HF1
Slug	1±0.1	0.6±0.1	1.8±0.1
TWIST1	1±0.5	4.5±0.7	13.2±2.6
ZEB1	1±0.2	0.6±0.5	0.5±0.2
ZEB2	1±0.5	0.2±0.1	1.9±0.9
GAPDH	1±0.1	1.1±0.1	1.1±0.1

Expression of transcriptional regulators of EMT according to Affymetrix Human Genome U133A microarrays. Results were normalized to the expression in the primary keratinocytes. GAPDH expression is given as internal control. Standard deviations are of three arrays for each cell type.

### Cell migration and Rac1 activity

**Figure 3:**
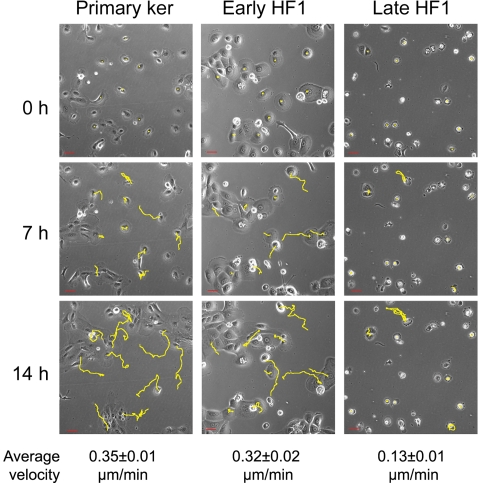
Individual cell motility in primary keratinocytes, early and late HF1 cells. Cell motility movies were created by acquiring live-cell images every 15 min throughout 14 h of the experiment. Cell tracks, in yellow, were determined by marking cell nuclei in every frame of the movie. The figure shows the same cells at the beginning of the experiment (0 h), after 7 h, and at the end of the movie (14 h). Cell velocity was calculated by marking the cell nucleus in every frame, and following cell movement. Average velocity was calculated by an application within the UCSF PRIISM environment ((http://msg.ucsf.edu/ive). Errors represent standard error of >100 cells examined in 3 independent experiments.

**Figure 4:**
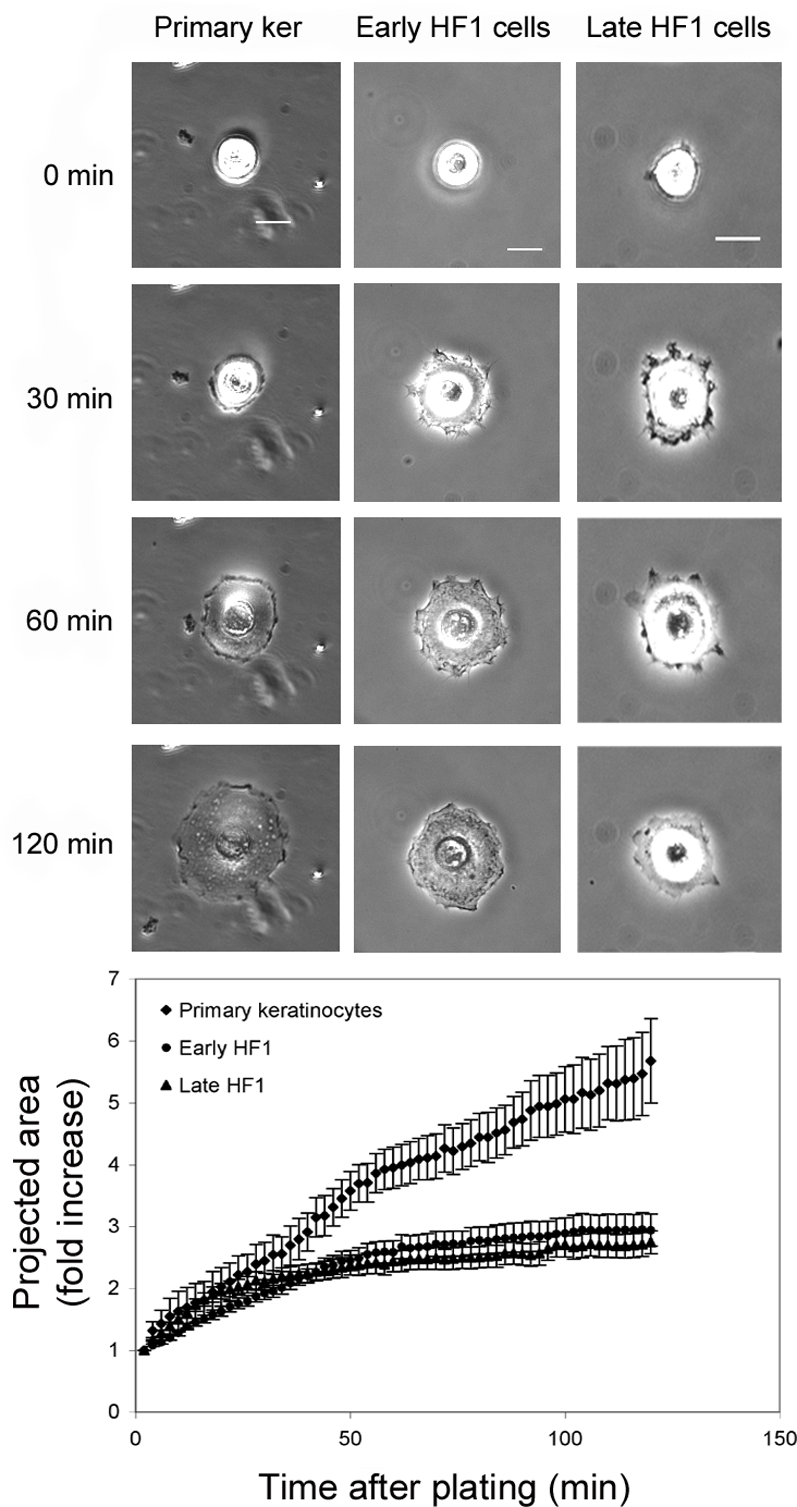
Cell spreading in the primary keratinocytes, early HF1 and late HF1 cells. Cell spreading movies were created by acquiring live-cell images every minute throughout 2 h of the experiment. A. Images show a representative cell in 4 time-points along the experiment, in the beginning (0 min), after 30 min, 60 min, and 120 min of the experiment. B. Graph shows quantification of cell area along the experiment. To quantify cell spreading, polygons, defining the cell perimeter were manually marked, at different time points, and the projected cell area was calculated by an application within the UCSF PRIISM environment (http://msg.ucsf.edu/ive). Error bars represent standard errors of >30 cells examined in 3 independent experiments.

One of the hallmarks of EMT is increased cell migration. We, on the other hand, noticed a marked reduction in the motility of cells during the progression from the primary keratinocytes and early HF1 to late HF1 cells. We examined individual cell motility by live-cell imaging, in which we followed live cells for 14 hours, acquiring phase contrast images every 15 min (Figure 3 and Movies S1, S2, S3). Tracking the migratory pathways, we calculated migration velocity for each cell type, based on randomly chosen cells (>100 cells). While the primary keratinocytes and early HF1 cells were highly motile (typical mean migration velocity: 0.35 µm/min), late HF1 cells barely moved throughout the experiment. Cell spreading, another feature that usually increases following EMT was also inhibited during the progression from the normal keratinocytes to late HF1 cells. We monitored cell spreading by acquiring live cell images every minute over two hours from cell plating until cells became fully spread on the plates. Figure 4A (and Movies S4, S5, S6) shows a representative cell, from each cell type at four time points starting before the beginning of attachment, to full spreading after 2 hours. Quantification of cell-spreading of ∼30 cells, showed that the highest spreading values were noted in the primary keratinocytes, and the lowest values were measured for late HF1 cells (Figure 4B). Early HF1 cells showed higher initial cell area, and the area of fully spread cells was similar to or higher than that of the primary keratinocytes (not shown), but the fold change is similar to that of late HF1 cells.

**Figure 5:**
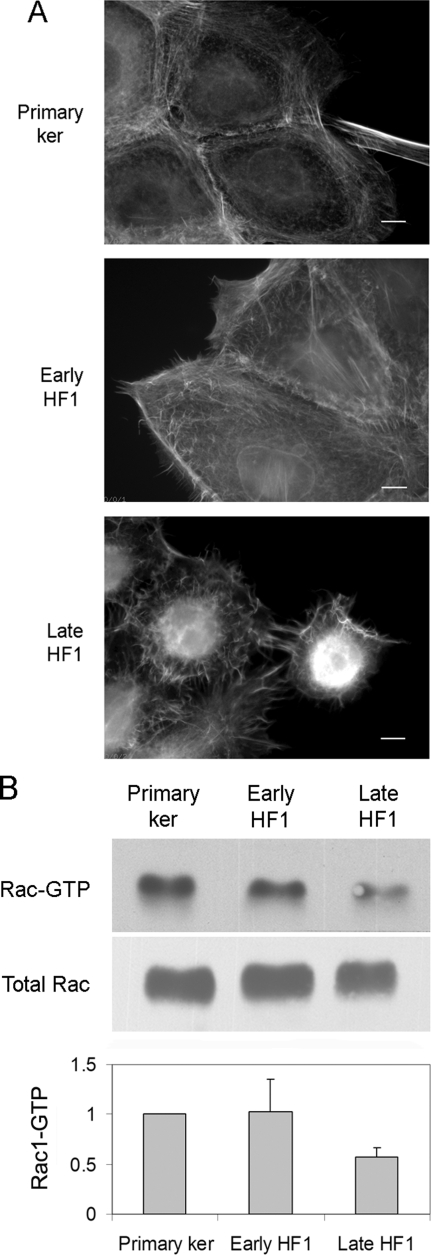
Disruption of Actin organization in late HF1 cells is associated with reduced Rac1 activity. A. Representative images of phalloidin staining for F-Actin in the primary keratinocytes, early and late HF1 cells. Bar = 15 µm. B. Western blot analysis of Rac1. Upper panel shows activated, GTP-loaded Rac1 pulled-down with GST-PBD. Lower panel shows total Rac1 levels in whole cell lysates before pull down. Graph shows average active, GTP-loaded Rac1. Error bars represent standard errors of three independent experiments.

Both cell spreading and migration are highly dependent on the dynamics of the actin cytoskeleton. Staining of the primary keratinocytes for F-actin, using phalloidin, showed ordered circumferential actin fibers forming lamellipodial protrusions from the cell surface (Figure 5A). Progression to early HF1 cells induced some disorder in actin organization, yet it did not affect the enrichment of actin along the cell periphery. A dramatic change occurred upon progression to late HF1 cells, which was characterized by poor actin organization, a marked absence of lamellipodial protrusions, and appearance of tangled microvilli (Figure 5A). Lamellipodia formation is known to be mediated primarily by the activity of the Rho-family GTPase Rac1. We therefore measured Rac1 activity by a pull-down assay, using PAK1 binding domain (PBD) fused to GST. We detected a 2-fold drop in Rac1 activity in late HF1 cells, compared to either the primary keratinocytes or early HF1 cells (Figure 5B).

**Figure 6:**
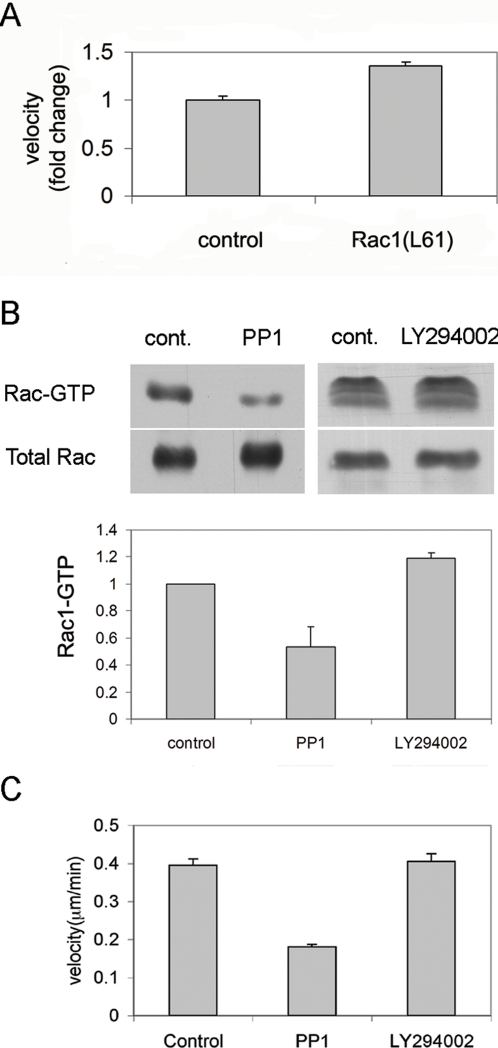
Src dependent Rac1 activity is essential for cell motility. A. Late HF1 cells were transfected with active Rac1 (Rac1L61) or with GFP as control. Cell motility movies were created by acquiring live-cell images every 15 min throughout 5 h of the experiment. Cell velocity was calculated by marking the cell nucleus in every frame, and following cell movement. Average velocity was calculated by an application within the UCSF PRIISM environment ((http://msg.ucsf.edu/ive). Graph shows fold change in velocity, normalized to the control cells. Errors represent standard error of >100 cells. B. Primary keratinocytes were treated with 5 µM PP1 or 5 µM LY294002 for 4 h before cell lysis and Rac1 activity assay. Graphs show quantification of Rac1-GTP. Error bars represent standard deviations of two experiments. C. Quantification of cell motility in the presence of PP1 or LY294002. Migration rate was examined as in A. Error bars represent standard error of >50 cells.

In order to examine whether low Rac1 activity in late HF1 cells is responsible for the reduced migration of these cells as compared to the primary keratinocytes, we transfected late HF1 cells with active-Rac (Rac1L61) and examined their migration velocity. Comparison of Rac1L61-transfected HF1 cells to GFP-transfected controls showed 1.4 fold increase in migration velocity (Figure 6A). When we consider that transfection efficiency was ∼30%, we can estimate a ∼2 fold increase in velocity as a result of active Rac1 expression. We further examined the signaling components, which may affect Rac1 activity. Rac1 is modulated by the activities of its exchange factors (GEFs) [22], which are regulated mainly by Src family kinases and by PI3K [22], [23]. In order to examine which of these kinases control Rac1 activity in our system, we examined the effect of the PI3K inhibitor LY294002 or of the Src inhibitor PP1 on Rac1 activity. Src inhibition with PP1 induced an over 2-fold decrease in Rac1 activity, whereas LY294002 had no apparent effect (Figure 6B). In agreement with these results, we found that PP1, but not LY294002, inhibited the migration of the primary keratinocytes (Figure 6C). Therefore Src activity in the primary keratinocytes is responsible for Rac1 activation and cell migration.

**Figure 7:**
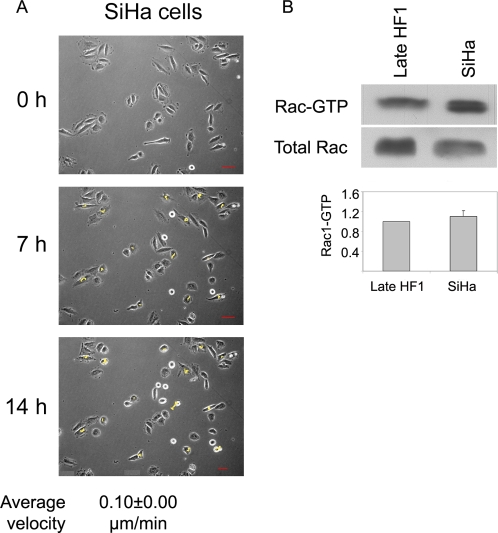
Migration and Rac1 activity in SiHa cells. A. Cell motility movies were created by acquiring live-cell images every 15 min throughout 14 h of the experiment. Cell tracks, in yellow, were determined by marking cell nuclei in every frame of the movie. The figure shows the same cells at the beginning of the experiment (0 h), after 7 h, and at the end of the movie (14 h). Cell velocity was calculated by marking the cell nucleus in every frame, and following cell movement. Average velocity was calculated by an application within the UCSF PRIISM environment ((http://msg.ucsf.edu/ive). Errors represent standard error of >200 cells. B. Western blot analysis of Rac1 in late HF1 cells and in SiHa cells. Upper panel shows activated, GTP-loaded Rac1 pulled-down with GST-PBD. Lower panel shows total Rac1 levels in whole cell lysates before pull down. Graph shows quantification of the active Rac1. Error bars represent standard errors of three independent experiments.

We further examined the generality of our findings, by examination of migration rate and Rac1 activity in the cervical cancer cell line SiHa. Live cell imaging of cells revealed that similar to late HF1 cells, SiHa cells hardly migrate during the 14 hours of the movie (Figure 7A and Movie S7). Quantification of cell motility showed average migration rate of 0.1 µm/min, which is even lower than the migration rate of late HF1 cells (0.13 µm/min). Furthermore, we found that Rac1 activity in SiHa cells was similar to the activity in late HF1 cells (Figure 7B). Of note, we detected reduced Rac1 protein level in SiHa cells compared to late HF1 cells, but the active fraction remained almost constant. We therefore show that in the course of transformation, up to the stage of tumor formation Rac1-dependent migration is reduced.

## Discussion

Morphological characterization of cervical dysplastic and cancerous tissues had been amply reported since the 1970's, showing a reduction in desmosomes and elevation in microvilli in cervical intraepithelial neoplastic lesions (CIN), as well as in cell culture models of HPV-transformed cells [10]–[13]. In the present study we integrated morphological data with microarray results and biochemical analysis, in order to gain insight into the cellular manifestations of early events in the transformation cascade. Interestingly, we observed an acquisition of a subset of EMT manifestations already at these early stages, but concomitantly, we monitored a gradual reduction in cell migration and in Rac1 activity.

In accordance with previous reports, we noted a marked reduction in desmosomes upon transition of primary keratinocytes through early HF1 cells to late HF1 cells (Figure 1 and Table 2). Alazawi et al showed reduced desmoglein expression in low-grade intraepithelial lesions [13], analogous to the decrease in desmosomal cadherins that we show in early HF1 cells (Table 2). Reductions in desmosomes in high-grade intraepithelial lesions [13] resemble the reductions we monitored in late HF1 cells, in desmogleins, desmocollins and other desmosomal components. Furthermore, immuno-staining and western blot of plakoglobin confirmed that the protein decreased significantly from the normal cells to early HF1 and to late HF1 cells (Figure 1B).

Down-regulation of desmosomes is associated with altered cytokeratin expression during cervical tumorigensis. Cytokeratins 1, 2, 10, 11, 13 and 16 are abundant in normal stratified epithelium [6], [24], equivalent to the primary keratinocytes used here. Our microarray results revealed a gradual reduction in cytokeratin 1, 4, 13, 15, 16, 23, 24 and 75, which initiated in early HF1 cells, and further decreased in late HF1 cells (Table 1). Elevation in cytokeratins 18 and 19, typical of simple epithelium, was dramatic, already in early HF1 cells. These results show that the loss of features of stratified epithelium, which is typical of cervical lesions, is initiated at the same time as cell immortalization with HPV16, and progresses with tumorigenesis.

Beyond the changes in the intermediate filament net, immense changes appeared in the actin cytoskeleton (Figure 5A), and in the associated adhesion complexes: adherens junctions and focal adhesions (Figure 2). Reduction in adherens junctions is a well-established characteristic of tumorigenesis, and we show here that the reduced expression of E-cadherin and decreased abundance of junctional beta-catenin occurs in the precancerous, late HF1 cells, (Figure 2A and Table 2). Nonetheless, changes in cell-matrix adhesion through focal adhesions and hemi-desmosomes during cervical cancer development are more controversial [25]–[27]. We report here that focal adhesions are reduced slightly upon transition from the primary keratinocytes to early HF1 cells and more extensively in the progression to late HF1 cells (Figure 2B). We found that down-regulation of adhesion preceded the ability of cells to survive and grow in an anchorage independent manner. This compelling observation suggests that in the course of transformation, cells adapt to grow in the absence of “survival signals” from the matrix or from neighboring cells. It seems that the reduced adhesion in early stages of transformation selects for those cells that do not depend on adhesion for their survival, leading to a progressive enrichment in anchorage independent cells in the pre-malignant cell population. Therefore the ability of transformed cells to grow in anchorage independent conditions may result from a stepwise selection process, driven by gradual reduction in cell adhesiveness.

We have shown that in late HF1 cells and in SiHa cells motility is dramatically reduced compared to the primary keratinocytes (Figures 3, 4 and 7, Movies S1, S2, S3, S4, S5, S6, S7). One of the known attributes of keratinoyctes is their ability to migrate, primarily for wound healing [28]. This feature of the normal cells was lost in the progression of transformation. Moreover, Rac1 activity in the various cell types correlated with migration capacity, and introduction of active Rac1 into late HF1 cells increased their motility. Our results showed that keratinocyte motility relied on their Src- dependent Rac1 activity (Figures 5 and 6). Rac1 is usually associated with a more transformed phenotype, since Rac1, through induction of lamellipodia formation, enables cells to dissociate, move from their original tissue, and translocate to a new destination [29]. Nonetheless we show here that Rac1 activity in immortalized and cancerous cells is down-regulated. Possibly, in metastatic cells Rac1 activity is regained and cells re-acquire migratory capacity.

There is an intricate interplay between adhesion complexes and Rac1, since Rac1 can trigger their assembly, but can also be activated by them. Rac1 plays a role in the formation of adherens junctions and has been shown to be essential for the recruitment of actin to E-cadherin [30]–[32]. Therefore, reduction in Rac1 activity from the primary keratinocytes to late HF1 cells may down-regulate adherens junction formation in the transformed cells. Furthermore, Rac1 is essential for recruitment of proteins to focal complexes [33], [34], therefore, low Rac1 activity in late HF1 cells may account for the reduced paxillin staining in focal adhesions. On the other hand, Rac1 may be activated in adhesion sites through Src and PI3K, which activate Rac1 GEFS. PI3K can be activated in adherens junctions [35]–[38] and in focal adhesions [39], [40], and activate PH-domain containing GEFS. Src, which is also activated in focal adhesions [40], [41], can phosphorylate p130Cas, which phosphorylates and activates the Rac1 GEF DOCK180 [42], [43], or can directly phosphorylate and activate the Rac1 GEFs Vav and Tiam1 [22], [23]. Further study is needed in order to fully understand the interactions between adhesion and signaling components in the complexes, among them the Rho family GTPases in our system.

In summary, in this study we show that very early in transformation, soon after HPV16 introduction, several features of EMT already emerge. These include: reduced desmosomes, keratins, adherens junctions and focal adhesions, with the concurrent, dramatic up-regulation of the transcription factor Twist. Nevertheless, in the anomalous EMT described here, Rac1 activity, as well as Rac1-dependent migration are reduced in both late HF1 cells and SiHa cells. We can therefore conclude that in these stages of transformation most epithelial features are eliminated. However, cells have not yet gained mesenchymal characteristics such as high vimentin expression, and have not acquired the motility and spreading typical of mesenchymal cells.

## Materials and Methods

### Cell culture and reagents

Primary keratinocytes and HF1 cells were grown in keratinocyte growth medium (67% DMEM, 23% HAM/F12, 10% FBS, 5 µg/ml insulin, 2 nM T3, 5 µg/ml transferrin, 0.4 µg/ml, hydrocortisone, 0.1 nM cholera toxin, 10 ng/ml EGF and antibiotics). Primary keratinocytes from second or third passage were used in the experiments reported here. HF1 cells were produced by transfection of the whole HPV16 genome into human foreskin keratinocytes [44], [45]. Early HF1 cells are ∼60 doublings and Late HF1 cells are ∼1000 doublings after HPV 16 transfection. SiHa cells were kindly provided to us by Professor S. Rosenbaum-Mitrani (Hadassah Hospital, Jerusalem, Israel). SiHa cells were grown in RPMI containing 10% FBS and antibiotics.

### Transmission electron microscopy (TEM)

Primary keratinocytes, early and late HF1 cells were grown in 10 cm plates until ∼70% confluence. Cells were fixed in Karnovsky's fixative (3% paraformaldehyde, 2% glutaraldehyde, 5 mM CaCl_2_ in 0.1 M cacodylate buffer pH 7.4, containing 0.1 M sucrose). Cells were scraped, pelleted and embedded in agar noble (final concentration of 1.7%), and postfixed with 1% OsO_4_, 0.5% potassium dichromate, and 0.5% potassium hexacyanoferrate in cacodylate buffer. The pellet was stained en bloc with 2% aqueous uranyl acetate, followed by ethanol dehydration, and embedded in Embed (EMS). Sections were cut, stained with 2% uranyl acetate in 50% ethanol and lead citrate, and examined using a CM12 (FEI Eindhoven, Holland) transmission electron microscope at an accelerating voltage of 120 kV. Digital images were obtained with a SIS Biocam CCD camera (FEI).

### Affymetrix microarrays

RNA from three independent cultures of primary keratinocytes and HF1 cells (early and late) was isolated using RNeasy kits (Qiagen, Germany). Approximately 10 µg of total RNA were reverse transcribed, amplified and labeled. 8 µg of cRNA were hybridized to Affymetrix Human Genome U133A microarrays, according to the manufacturer's protocol. The signal of each array was scaled to the intensity value of specific constant controls. The expression value of each gene in early and late HF1 cells was normalized to the expression level in the primary keratinocytes.

### Fluorescence microscopy

HF1 cells and primary keratinocytes were plated on fibronectin-coated coverslips in 24-well plates at a concentration of 3×10^4^ cells per well and 5×10^4^ cells per well respectively. 48 h after plating, cells were fixed with 3% paraformaldehyde containing 0.5% TritonX100 for 30 sec followed by incubation with 3% paraformaldehyde for 30 min. Cells were stained with phalloidin-FITC from Sigma-Aldrich (Israel) for actin, or immunolabelled with anti-beta-catenin, anti-plakoglobin or anti-paxillin from BD-Transduction (USA). Images were acquired with a DeltaVision system (Applied Precision Inc., Issaquah, WA, USA) equipped with an inverted microscope IX70 (Olympus, Tokyo, Japan).

### Immuno-blotting

Equal amounts of protein from samples of the primary keratinocytes, early and late HF1 cells were separated by SDS-PAGE and analyzed by western blot. The antibodies used were: Anti-Plakoglobin and anti-Rac1 from Transduction laboratories.

### Rac1 activity assay

Preparation of GST-PBD beads: GST-PBD (PAK binding domain) was expressed in BL21 E.Coli. After induction, the bacteria were suspended in a buffer containing 20 mM HEPES pH7.5, 120 mM NaCl, 10% glycerol, 2 mM EDTA and protease inhibitors, sonicated, and centrifuged. NP40 was added to a final concentration of 0.5% before loading it on glutathione-sepharose beads. The lysate was incubated with the beads for 1 h at 4°C, followed by 5 washes with NP40-containing lysis buffer.

Rac1 binding: Primary keratinocytes, HF1 cells and SiHa cells were grown in 10 cm plates to 30% confluence, lysed with a buffer containing 50 mM Tris pH8.0, 0.5 M NaCl, 1% NP40, 0.5% deoxycholate, 0.1% SDS, 10 mM MgCl_2_ and protease inhibitors, sonicated and centrifuged. 1 mg of each sample was incubated with the GST-PBD beads for 1 h, to allow binding of active, GTP-bound Rac1 to the PBD, followed by five washes with a buffer containing 20 mM HEPES, 120 mM NaCl, 1% NP40 10 mM MgCl_2_ and protease inhibitors. Samples were separated by SDS-PAGE and compared to samples of whole cell lystaes. Western blots were reacted with an anti-Rac1 antibody (Transduction laboratories).

### Live-cell imaging

For acquisition of motility movies, HF1 cells, SiHa cells and primary keratinocytes were plated on fibronectin-coated 24-well plates. Images were automatically acquired every 15 min throughout 14 h of the experiment, with a DeltaVision system (Applied Precision Inc.). Quantification of cell velocity was performed by marking the cell nucleus in every frame, following cell movement. The imaging software was written as an application within the UCSF PRIISM environment (http://msg.ucsf.edu/ive).

For acquisition of cell spreading movies, HF1 cells and the primary keratinocytes were plated on fibronectin-coated glass-bottom 35 mm plates at a concentration of 30,000 cells per plate. Spreading movies initiated 15 min after cell-plating, and images were taken every minute for 2 hours. To quantify cell spreading, polygons, tracing the cell perimeter were manually marked every second time point, and the projected cell area was calculated. The imaging software was written as an application within the UCSF PRIISM environment (http://msg.ucsf.edu/ive).

### Transfection

Late HF1 cells were plated on fibronectin-coated 24-well plates at a concentration of 40000 cells per well. One day after plating cells were transfected with pCB6-Rac1L61-GFP or with E-GFP as control. Transfection was performed with SAINT-MIX (Synvolux Therapeutics B.V.) according to the manufacturer instructions. One day after transfection cells were trypsinized and 20000 cells were re-plated to enable examination of cell motility in sparse cultures. Live cell imaging of cultures was performed as described above.

## Supporting Information

Movie S1 Live-cell imaging of keratinocyte motility. Cell motility movies were created by acquiring live-cell images every 15 min throughout 14 h of the experiment. Bar = 50 µm.
(1.53 MB MOV)

Movie S2 Live-cell imaging of early HF1 motility. Cell motility movies were created by acquiring live-cell images every 15 min throughout 14 h of the experiment. Bar = 50 µm.
(1.58 MB MOV)

Movie S3 Live-cell imaging of late HF1 motility. Cell motility movies were created by acquiring live-cell images every 15 min throughout 14 h of the experiment. Bar = 50 µm.
(1.29 MB MOV)

Movie S4 Live cell imaging of keratinocyte spreading. Cell spreading movies were created by acquiring live-cell images every minute throughout 2 h of the experiment. Bar = 50 µm.
(2.52 MB MOV)

Movie S5 Live cell imaging of early HF1 spreading. Cell spreading movies were created by acquiring live-cell images every minute throughout 2 h of the experiment. Bar = 50 µm.
(2.08 MB MOV)

Movie S6 Live cell imaging of late HF1 spreading. Cell spreading movies were created by acquiring live-cell images every minute throughout 2 h of the experiment. Bar = 50 µm.
(1.97 MB MOV)

Movie S7 Live-cell imaging of SiHa cells motility. Cell motility movies were created by acquiring live-cell images every 15 min throughout 14 h of the experiment. Bar = 50 µm.
(2.28 MB MOV)
